# Treatment patterns, effectiveness, and safety of daratumumab-based regimens in Chinese patients with multiple myeloma: longer follow-up of the real-world MMY4032 study

**DOI:** 10.1007/s00277-026-06972-8

**Published:** 2026-04-13

**Authors:** Wei Yang, Luqun Wang, Yafei Wang, Ting Niu, Rong Fu, Yuping Zhong, Wenbin Qian, Kaiyang Ding, Kai Sun, Hong Liu, Baijun Fang, Hui Liu, Yanhui Li, Yishen Yang, Jianmin Zhuo, Xi Chen, Bijie Xun, Jin Lu

**Affiliations:** 1 Shengjing Hospital of China Medical University, Liaoning, China; 2 Qilu Hospital of Shandong University, Shandong, China; 3 Tianjin Medical University Cancer Institute and Hospital, National Clinical Research Center for Cancer, Tianjin’s Clinical Research Center for Cancer, Tianjin, China; 4 West China Hospital Sichuan University, Sichuan, China; 5 Tianjin Medical University General Hospital, Tianjin, China; 6 Qingdao Municipal Hospital, Qingdao, Shandong China; 7 Second Affiliated Hospital of Zhejiang University, Zhejiang, China; 8 First Affiliated Hospital of University of Science and Technology of China, Anhui, China; 9 Henan Provincial People’s Hospital, Henan, China; 10 Affiliated Hospital of Nantong University, Jiangsu, China; 11 Henan Cancer Hospital, Henan, China; 12 Beijing Hospital, Beijing, China; 13 Johnson & Johnson, Beijing, China; 14 IQVIA, Shanghai, China; 15 Johnson & Johnson, Shanghai, China; 16 Peking University People’s Hospital, National Clinical Research Center for Hematologic Disease, No.11 Xizhimen South Street, XiCheng District, Beijing, 100044 China; 17 Collaborative Innovation Center of Hematology, Soochow, China

**Keywords:** Daratumumab, Real-world evidence, Multiple myeloma, Clinical outcomes

## Abstract

The multicenter, noninterventional, observational MMY4032 study is a large-scale, real-world study exploring daratumumab (DARA) in Chinese patients (*n* = 212) with multiple myeloma (MM) who have received ≤ 3 prior lines of therapy. In the first interim analysis (median follow-up, 10.5 months), DARA was often initiated in second-line therapy, often given in combination with a proteasome inhibitor and/or immunomodulatory drug, and induced high response rates (overall response rate [ORR], 71.8%) and acceptable safety. Here, we report treatment patterns, efficacy, and safety from the second interim analysis with a longer median follow-up of 16.2 months. At the time of this analysis, median duration of DARA exposure was 8.2 months. Among 189 response-evaluable patients, the ORR was 74.1% and the very good partial response or better rate was 55.6%. Minimal residual disease–negativity rate was 60.0% among tested patients. Median progression-free and overall survival were 32.8 months and not reached, respectively; 12-month rates were 77.9% and 87.8%. Response and survival rates were higher with DARA initiation in earlier lines of therapy. Median time to next treatment was not reached with most DARA-based regimens. Adverse drug reactions and serious adverse events were reported in 20.3% and 15.6% of patients, respectively. Overall, with longer follow-up of the MMY4032 study, responses deepened, and survival rates remained high with DARA-based regimens, with more favorable outcomes observed with earlier DARA initiation. No new safety concerns were observed. These real-world data continue to support early use of DARA-based regimens as a standard of care for Chinese patients with MM.

**Supplementary Information:**

The online version contains supplementary material available at 10.1007/s00277-026-06972-8.

## Introduction

Daratumumab (DARA), a human IgGκ monoclonal antibody targeting CD38 with a direct on-tumor [1–4] and immunomodulatory [5–7] mechanism of action, has demonstrated promising efficacy in global populations of patients with newly diagnosed multiple myeloma (NDMM) and relapsed or refractory multiple myeloma (RRMM) [8–11]. In China, DARA was first approved in 2019 as a monotherapy in patients with RRMM and has since gained additional approval in 2021 in combination with lenalidomide and dexamethasone (D-Rd) and bortezomib and dexamethasone (D-Vd) for patients with RRMM, and with lenalidomide and dexamethasone (D-Rd) and bortezomib, melphalan, and prednisone (D-VMP) for transplant-ineligible patients with NDMM.

OCTANS and LEPUS are phase 3 randomized clinical trials (RCTs) that investigated the efficacy and safety of D-VMP and D-Vd, respectively, in an Asian patient population. D-VMP and D-Vd led to deep responses and improved progression-free survival (PFS) versus bortezomib, melphalan, and prednisone (VMP) and bortezomib and dexamethasone (Vd) alone in transplant-ineligible Asian patients with NDMM (PFS hazard ratio [HR], 0.43; 95% confidence interval [CI], 0.24–0.77; *P* = 0.0033) and Chinese patients with RRMM (PFS HR, 0.35; 95% CI, 0.24–0.51; *P* < 00001), respectively [12, 13]. Furthermore, DARA was well tolerated in these populations, and no new safety concerns were observed.

The large-scale, multicenter, observational MMY4032 study was designed to explore the real-world use of DARA in Chinese patients with multiple myeloma (MM) in routine clinical practice, which will serve, in part, to provide important information to support RCT data [14]. Results from the first MMY4032 interim analysis, with a median follow-up of 10.5 months, revealed that Chinese patients with MM were diagnosed at a relatively young age and with a higher prevalence of advanced disease compared with other global regions [14]. The majority of patients had received ≥ 1 line of therapy prior to DARA initiation, and most patients had prior exposure to a proteasome inhibitor (PI) and/or an immunomodulatory drug (IMiD). Chinese patients were found to commonly initiate DARA-based regimens as a second-line treatment, which is not unexpected given that the study was initiated just after DARA was approved for frontline use in China, and often in combination with PIs or IMiDs; the most common of each combination was DARA plus Vd and DARA plus pomalidomide/dexamethasone, respectively. While favorable clinical outcomes and safety profiles were observed in the first interim analysis, the relatively short follow-up time limited the evaluation of long-term outcomes and restricted comprehensive comparisons. Additional follow-up was needed to provide further data on subsequent therapy use following DARA treatment, allowing for a more in-depth understanding of treatment patterns, as well as more mature response and survival data, permitting more valuable inferences to be made.

Herein, we present extended follow-up data from the MMY4032 real-world effectiveness and safety study of Chinese patients with MM who were treated with DARA, including results based on various DARA-based regimens and lines of therapy and across different patient subgroups. Notably, the overall MRD-negativity rate observed in Chinese clinical practice was evaluated. Additionally, the longer follow-up period will support the analysis of time to non-DARA subsequent therapies.

## Methods

### Study design and patients

This is an ongoing, multicenter, noninterventional, observational study across 13 participating sites in China; the full study design and eligibility criteria have been previously published [14]. Briefly, eligible patients were ≥ 18 years of age, had symptomatic NDMM or RRMM, and had either started DARA after August 1, 2019, and were to continue DARA at the time of study initiation (November 3, 2021) or started DARA after study initiation. The decision to treat with DARA must have been made prior to and independently of the patient’s inclusion in the study, and treatment was administered in accordance with local clinical practice. Patients who had received ≥ 4 prior lines of MM therapy before starting DARA-based treatment, who had a diagnosis of other cancers, or who were currently participating in another investigational study, were excluded.

### Data collection

For patients who started DARA after August 1, 2019, but before study initiation, data were collected retrospectively using medical chart reviews; data were collected prospectively thereafter. For patients who started DARA after study initiation, data were collected prospectively. Prospective data collection was intended for every 2 months within the first 12 months after enrollment and every 6 months thereafter until the end of the study. Baseline was defined as the latest status prior to the first dose of DARA within the study.

### Study assessments and endpoints

Primary and secondary endpoints have been previously published with the first interim analysis (data cutoff date: April 30, 2023) [14] and are summarized in the Supplementary Methods.

With a longer follow-up of 16.2 months, the second interim analysis reported here provides data on treatment response (per the International Myeloma Working Group [IMWG] response criteria), minimal residual disease (MRD) negativity, survival outcomes (PFS and overall survival [OS]), and time to next treatment (TTNT). MRD testing results were collected from the case report forms and were categorized as MRD positive, MRD negative, or “other.” The sensitivity of MRD assays generally ranged from 10–^4^ to 10–^6^. Additional safety data on outcomes such as adverse drug reactions (ADRs) and serious treatment-emergent adverse events (TEAEs) were also collected throughout the extended follow-up period to allow for further observation of DARA safety in the real-world setting. Response rates, PFS, and OS were evaluated overall and based on specific type of DARA-based regimen, treatment line in which DARA was initiated, and in various patient subgroups based on age, revised cytogenetic risk status, and treatment duration. Revised high cytogenetic risk was defined as ≥1 of the following high-risk cytogenetic abnormalities: t(4;14), t(14;16), del(17p), t(14;20), gain(1q21), and amp(1q21). TTNT was evaluated overall and based on type of DARA-based regimen and by line of therapy in which DARA was initiated. MRD negativity and safety were evaluated in the overall population.

### Statistical analyses

This second interim analysis (12 months after the last patient was enrolled) was performed using a cutoff date of December 1, 2023. Full statistical methods have been previously reported [14]. Clinical outcomes were analyzed during the DARA-based treatment period, which is defined as the time between initiation of DARA and commencement of next non-DARA therapy line. Note that OS was defined as the time from the initiation of DARA to the date of death (with death due to any cause), and patients were censored at the last known date to be alive. Treatment response and MRD negativity were assessed in the response-evaluable population, defined as all patients who received ≥ 1 dose of DARA and had ≥ 1 tumor response evaluation, disease progression, or death. Survival outcomes and safety were based on the safety-analysis set, defined as all patients who received ≥ 1 dose of DARA. Continuous and categorical variables were summarized using descriptive statistics, and the Kaplan-Meier method was used to estimate time-to-event variables.

## Results

### Patient characteristics

As of the cutoff date, 212 patients who had a diagnosis of MM and received ≥ 1 DARA treatment after August 1, 2019, were eligible for this second interim analysis. Full patient demographics and clinical characteristics have been previously reported [14] and are summarized in Supplementary Table 1. Overall, baseline characteristics were generally similar across DARA-based regimens. Among patients who received DARA + PI ± dexamethasone, the frequency of International Staging System stage III disease was high (*n* = 22/36 [61.1%]). As of data cutoff, 113 (53.3%) patients were ongoing in the study, and 99 (46.7%) patients had discontinued due to withdrawal (*n* = 51), death (*n* = 33), participation in another trial (*n* = 8), and loss to follow-up (*n* = 7).

### Treatment duration and exposure

Detailed information on treatment patterns have been previously published with a median follow-up of 10.5 months [14]. With a longer median (range) follow-up of 16.2 (0-50.6) months, duration of DARA exposure increased for the overall population with a median duration of 8.2 (0-49.1) months. As observed in the first interim analysis, duration of DARA exposure remained longer when DARA was initiated in earlier lines of therapy (first line, 9.7 [0.3–37.0]; second line, 8.1 [0-49.1]; third line, 7.5 [0.1–31.5]; fourth line, 4.7 [0.5–28.7] months; Supplementary Table 2). For the most common regimen types, DARA + IMiD ± dexamethasone and DARA + PI ± dexamethasone, median (range) duration of DARA exposure was 8.1 (0–37.0) months and 8.5 (0.3–27.7) months, respectively.

### Daratumumab treatment effectiveness

**Fig. 1:**
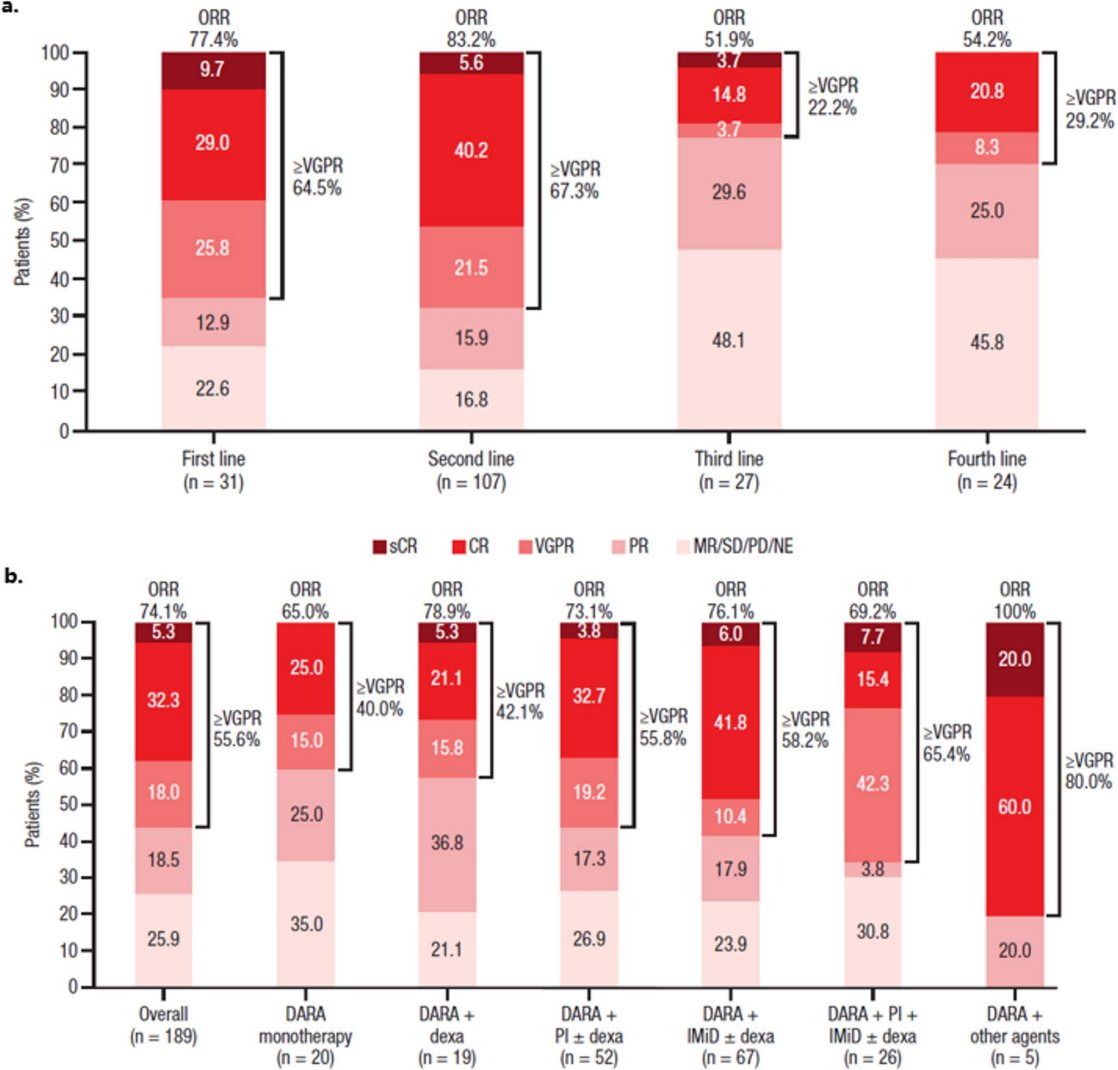
Treatment response by (**a**) treatment line in which DARA was initiated^a^ and (**b**) DARA-based regimen CR, Complete response; DARA, Daratumumab; dexa, Dexamethasone; IMiD, Immunomodulatory drug; MR, Minimal response; NE, Not evaluable; ORR, Overall response rate; PD, Progressive disease; PI, Proteasome inhibitor; PR, Partial response; sCR, Stringent complete response; SD, Stable disease; VGPR, Very good partial response. ^a^In patients with available post-DARA disease assessment

Among the 189 (89.2%) patients included in the response-evaluable set, ORR was 74.1%, and the rate of very good partial response or better (≥ VGPR) was 55.6%. Overall, higher response rates were observed when DARA was initiated in earlier lines of therapy, both for ORR (first line, 77.4%; second line, 83.2%; third line, 51.9%; fourth line, 54.2%) and ≥VGPR (first line, 64.5%; second line, 67.3%; third line, 22.2%; fourth line, 29.2%; Fig. 1a). Of the 2 most common DARA-based regimens identified, DARA + IMiD ± dexamethasone and DARA + PI ± dexamethasone, ORRs were comparable at 76.1% and 73.1%, respectively. The rate of ≥VGPR was also comparable for patients receiving DARA + PI ± dexamethasone (55.8%) and DARA + IMiD ± dexamethasone (58.2%), but was higher in those receiving DARA + PI + IMiD ± dexamethasone (65.4%) (Fig. 1b). ORRs in patient subgroups were generally consistent with the overall response-evaluable set, but with higher response rates in patients with International Staging System stage I disease (85.2%) and those with revised high cytogenetic risk (82.9%; Supplementary Fig. 1).

A total of 60 patients underwent MRD testing after initiation of DARA, with 52 (86.7%) patients tested using flow cytometry. The overall MRD-negativity rate (with ≥1 MRD-negative result throughout the study) was 60.0% (36/60), while the MRD-negativity rate during the initiation of DARA up to the end of DARA use was 48.3% (29/60; Table 1). Additionally, among the 29 patients who achieved MRD negativity during treatment with a DARA-based regimen, only 2 patients converted to MRD positivity after achieving MRD negativity. Among the 60 patients who underwent MRD testing, the median time to the first MRD-negative result was 7.6 months, with an estimated 12-month time to first MRD-negativity rate of 61.1% (Table 1).

**Table 1:** Summary of MRD negativity

	Overall (*N* = 60)^a, b^
MRD-negativity rate at any time during the study, *n* (% [95% CI])	
At any time during the study	36 (60.0 [46.5–72.4])
During initiation of DARA up to end of DARA use	29 (48.3 [35.2–61.6])
Time to first MRD negativity	
Median (95% CI), mo	7.6 (6.1-NE)
12-mo time to first MRD negativity (95% CI), %	61.1 (42.5–73.7)

*CI* Confidence interval, *DARA* Daratumumab, *MRD* Minimal residual disease

^a^In patients with available post-DARA disease assessment who underwent MRD testing

^b^Among patients in the overall population who underwent testing (*n* = 60), MRD testing was performed at a sensitivity threshold of 10^–4^ (31.7%), 10^–5^ (40.0%), 10^–6^ (8.3%), or other (20.0%). Data of positive, negative, and other were collected directly from the raw data

**Fig. 2:**
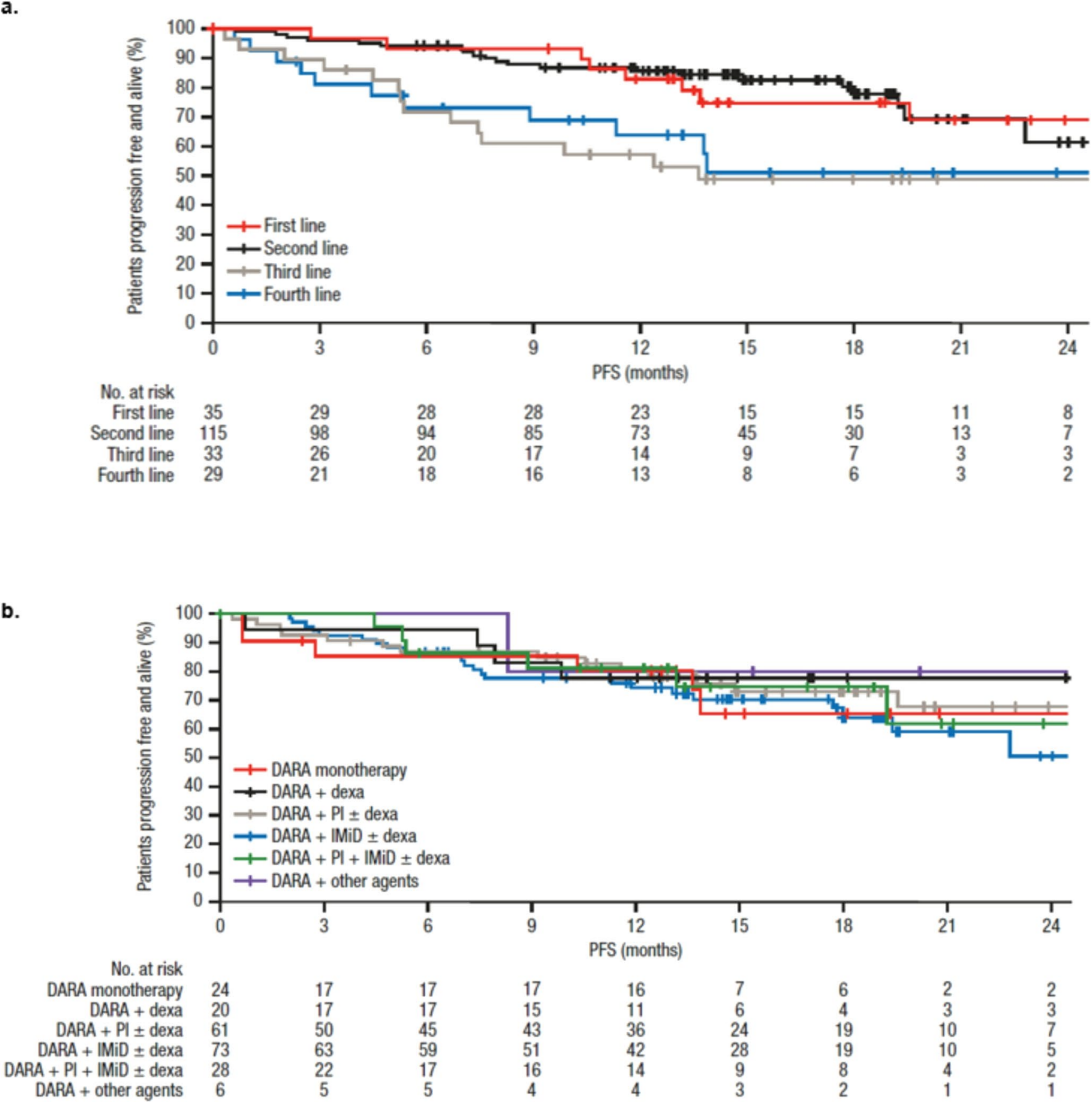
PFS by (**a**) treatment line in which DARA was initiated^a^ and (**b**) DARA-based regimen DARA, Daratumumab; dexa, Dexamethasone; IMiD, Immunomodulatory drug; PFS, Progression-free survival; PI, Proteasome inhibitor

Median PFS had been reached at the time of the current analysis and was 32.8 (95% CI, 27.1-not estimable) months, with an estimated 12-month PFS rate of 77.9%. Median OS was still not reached, with an estimated 12-month OS rate of 87.8%. Greater survival benefits were observed when DARA was initiated in earlier lines of therapy, for both estimated 12-month PFS and OS rates (Table 2; Fig. 2a). While patients who received DARA in the first or second line were categorized as the early line group whereas those treated in the third or fourth line were defined as the late line group, obvious 12-month PFS benefits were observed in early line group compared with late line group (85.1% vs. 60.6%, *p* < 0.0001) (Supplementary Fig. 2a).

**Table 2:** PFS and OS by line of therapy in which DARA was initiated and DARA-based regimen

Survival outcome	Overall (*N* = 212)	First line (*n* = 35)	Second line (*n* = 115)	Third line (*n* = 33)	Fourth line (*n* = 29)		
PFS							
Median (95% CI), mo	32.8(27.1-NE)	NE(19.6-NE)	27.1(22.8-NE)	13.6(6.7-NE)	NE(8.9-NE)		
12-mo PFS (95% CI), %	77.9 (71.1-83.3)	83.0 (63.8-92.5)	85.7 (77.1-91.3)	57.5 (37.4-73.2)	64.0 (41.8-79.6)		
OS							
Median (95% CI), mo	NE(NE-NE)	NE(26.0-NE)	NE(NE-NE)	NE(NE-NE)	NE(13.9-NE)		
12-mo OS (95% CI), %	87.8 (82.3-91.7)	97.1 (81.4-99.6)	90.4 (82.7-94.7)	76.4 (56.5-88.0)	78.6 (58.3-89.8)		

Survival outcome	Overall (N = 212)	DARA monotherapy (n = 24)	DARA + dexa(n = 20)	DARA + PI ± dexa(n = 61)	DARA + IMiD ± dexa(n = 73)	DARA + PI + IMiD ± dexa(n = 28)	DARA + other agents (n = 6)
PFS							
Median (95% CI), mo	32.8(27.1-NE)	NE(13.6-NE)	NE(NE-NE)	NE(19.6-NE)	27.1(18.0-NE)	32.8(13.2-NE)	NE(8.3-NE)
12-mo PFS (95% CI), %	77.9 (71.1-83.3)	80.4 (55.8-92.2)	77.8 (51.1-91.0)	80.6 (66.8-89.1)	74.3 (61.9-83.2)	81.3 (57.4-92.6)	80.0 (20.4-96.9)
OS							
Median (95% CI), mo	NE(NE-NE)	NE(NE-NE)	NE(13.3-NE)	NE(26.0-NE)	NE(NE-NE)	NE(23.8-NE)	NE(8.3-NE)
12-mo OS (95% CI), %	87.8 (82.3-91.7)	87.3 (65.6-95.7)	84.1 (58.3-94.6)	88.1 (76.7-94.2)	86.3 (75.3-92.7)	96.3 (76.5-99.5)	83.3 (27.3-97.5)

*CI* Confidence interval, *DARA* Daratumumab, *dexa* Dexamethasone, *IMiD* Immunomodulatory drug, *NE* Not estimable, *OS* Overall survival, *PFS* Progression-free survival, *PI* Proteasome inhibitor

The highest estimated 12-month PFS and OS rates were observed in patients receiving DARA + PI + IMiD ± dexamethasone, 81.3% and 96.3%, respectively (Table 2; Fig. 2b). Daratumumab treatment in earlier lines of therapy is of particular interest. Among patients who initiated daratumumab in early lines, progression‑free survival appeared broadly comparable across different daratumumab‑based regimens, with no clear differences observed (Supplementary Fig. 2b). Several clinical factors were also evaluated, including age and treatment duration. PFS and OS were similar between patient subgroups based on age (< 70 years vs. ≥70 years) and revised cytogenetic risk (standard vs. high; Supplementary Fig. 3 and Supplementary Fig. 4). Patients who received a longer duration of DARA therapy had prolonged PFS (treatment duration ≥12 vs. < 12 months: *P* < 0.0001; treatment duration ≥18 vs. < 18 months: *P* = 0.0002; Supplementary Fig. 3) and OS (treatment duration ≥12 vs. < 12 months: *P* = 0.0003; treatment duration ≥18 vs. < 18 months: *P* = 0.013; Supplementary Fig. 4).

### Subsequent MM therapy

In the current analysis of TTNT (defined as the interval from DARA initiation to switching to next line of therapy due to lack of efficacy, disease progression, or death), median TTNT was not reached across most subgroups based on line of therapy in which DARA was initiated and most DARA-based regimens (Table 3). The estimated proportion of patients free from next line of treatment at 12 months for the overall population was 82.1%.

**Table 3:** TTNT by line of therapy in which DARA was initiated and DARA-based regimen and subsequent therapy by DARA-based regimen

n (%)	Overall (N = 212)		First line(n = 35)	Second line(n = 115)	Third line(n = 33)	Fourth line(n = 29)	
TTNT^a^							
Median (95% CI)	NE(32.8-NE)		NE(23.1-NE)	NE(22.8-NE)	32.8(10.0-NE)	NE(11.3-NE)	
Proportion of patients free from next line of treatment at 12 mo, %	82.1		94.3	86.8	62.0	68.7	

	Overall (N = 212)	DARA monotherapy (n = 24)	DARA + dexa(n = 20)	DARA + PI ± dexa(n = 61)	DARA + IMiD ± dexa(n = 73)	DARA + PI + IMiD ± dexa(n = 28)	DARA + other agents (n = 6)
TTNT^a^							
Median (95% CI), mo	NE(32.8-NE)	NE(17.7-NE)	NE(NE-NE)	NE(23.1-NE)	NE(19.4-NE)	32.8(23.3-NE)	NE(8.3-NE)
Proportion of patients free from next line of treatment at 12 mo, %	82.1	87.3	78.8	84.5	77.5	87.5	83.3

	Overall (N = 212)	DARA monotherapy (n = 24)	DARA + dexa(n = 20)	DARA + PI ± dexa(n = 61)	DARA + IMiD ± dexa(n = 73)	DARA + PI + IMiD ± dexa(n = 28)	DARA + other agents (n = 6)
Subsequent therapy,^b^ n (%)	51 (24.1)	2 (8.3)	5 (25.0)	13 (21.3)	21 (28.8)	10 (35.7)	0
Most common (≥5 patients)							
KPd	7 (3.3)	0	0	1 (1.6)	4 (5.5)	2 (7.1)	0
P	6 (2.8)	0	0	0	6 (8.2)	0	0
KCd	5 (2.4)	0	0	1 (1.6)	3 (4.1)	1 (3.6)	0
R	5 (2.4)	0	0	1 (1.6)	1 (1.4)	3 (10.7)	0
Reason for subsequent therapy^c^							
n	51	2	5	13	21	10	0
Physician recommendation	38 (74.5)	1 (50.0)	4 (80.0)	10 (76.9)	14 (66.7)	9 (90.0)	0
Disease progression	17 (33.3)	2 (100)	0	2 (15.4)	9 (42.9)	4 (40.0)	0
Patient request	2 (3.9)	0	1 (20.0)	0	1 (4.8)	0	0
Unknown	7 (13.7)	0	2 (40.0)	3 (23.1)	0	2 (20.0)	0

*CI* Confidence interval, *DARA* Daratumumab, *dexa* Dexamethasone, *IMiD* Immunomodulatory drug, *KCd* Carfilzomib/cyclophosphamide/dexamethasone, *KPd* Carfilzomib/pomalidomide/dexamethasone, *NE* Not estimable, *P* Pomalidomide, *PI* Proteasome inhibitor, *R* Lenalidomide, *TTNT* Time to next treatment

^a^TTNT events were defined as lack of efficacy, disease progression, or death

^b^Percentages are based on the total number of patients in each respective treatment group

^c^Multiple reasons may have been provided for each patient

A total of 51 of 212 (24.1%) patients went on to receive non-DARA subsequent therapies (Table 3), the most common (≥ 5 patients overall) of which were carfilzomib/pomalidomide/dexamethasone (*n* = 7/51 [13.7%]), pomalidomide (*n* = 6/51 [11.8%]), carfilzomib/cyclophosphamide/dexamethasone (*n* = 5/51 [9.8%]), and lenalidomide (*n* = 5/51 [9.8%]). Of the 51 patients who initiated subsequent non-DARA therapy, the primary reason for doing so was physician recommendation (*n* = 38/51 [74.5%]) followed by disease progression (*n* = 17/51 [33.3%]) (Table 3). Considering the distribution of primary reasons, not all subsequent therapies were initiated due to disease progression; rather, these were more often initiated due to physician recommendation under real-world clinical practice.

### Safety

With additional follow-up from the first interim analysis, no new safety concerns were observed (Table 4). In the overall safety population, ADRs were reported in 43 of 212 (20.3%) patients. The most common ADRs (≥ 5% of patients) were leukopenia (8.0%), neutropenia (7.1%), lymphopenia (5.7%), and hypogammaglobulinemia (5.2%). Serious TEAEs were reported in 33 (15.6%) patients. Of all serious TEAEs, infections and infestations (*n* = 20/212 [9.4%]) remained the most common group, consisting primarily of pneumonia, which remained the only serious TEAE reported in ≥ 5 patients overall (5.7%), and COVID-19 (1.9%). Overall, 29 (13.7%) patients experienced TEAEs that led to discontinuation of DARA-based treatment. As of the cutoff date, a total of 33 patients had died. Primary causes of death were progressive disease (*n* = 12) and unknown due to the family declining to disclose the reason or cause of death (*n* = 13). A total of 8 deaths were due to adverse events; however, only 1 was considered related to DARA (acute onset chronic liver failure).

**Table 4:** Safety outcomes by overall population^a^

Safety outcome, *n* (%)	Overall (*N* = 212)
ADRs	43 (20.3)
Events in ≥ 5 patients^b^	
Leukopenia	17 (8.0)
Neutropenia	15 (7.1)
Lymphopenia	12 (5.7)
Hypogammaglobulinemia	11 (5.2)
Serious TEAEs	33 (15.6)
Events in > 1 patient^b^	
Infections	20 (9.4)
Pneumonia	12 (5.7)
COVID-19	4 (1.9)
No. of patient-reported TEAEs leading to discontinuation	29 (13.7)
Deaths	33 (15.6)
Primary cause of death^c^	
Disease progression	12 (36.4)
AE	8 (24.2)
Other/unknown^d^	13 (39.4)

*ADR* Adverse drug reaction, *AE* Adverse event, *DARA* Daratumumab, *TEAE* Treatment-emergent adverse event

^a^ADRs and TEAEs were collected before the next non-DARA therapy line, while deaths were collected during the entire study period

^b^Events occurring in the indicated number of patients within any treatment group

^c^Percentages calculated using the number of deaths as the denominator

^d^Unknown due to family choosing not to disclose reason or cause of death

## Discussion

The MMY4032 study was designed to gain an understanding of treatment patterns and clinical outcomes of Chinese patients with MM who were treated with DARA in routine clinical practice. While the primary focus of the first interim analysis was to identify and describe treatment patterns, the relatively short follow-up period at the time of that analysis limited the data on treatment response. In the current analysis, with a longer median follow-up of 16.2 months, the data are more mature, thereby allowing valuable inferences to be made regarding long-term clinical outcomes in this real-world patient population. With additional follow-up, responses continued to deepen across DARA-based regimens, with an ORR of 74.1% and ≥VGPR rate of 55.6%. Median PFS was reached (32.8 months), with notable estimated 12-month rates of 77.9% for PFS and 87.8% for OS. PFS and OS were similar between patient subgroups based on age and revised cytogenetic risk, but patients who received a longer duration of DARA therapy had prolonged PFS and OS. Median TTNT was not reached with the majority of DARA-based regimens, and overall, the proportion of patients free from next line of treatment at 12 months was 82.1%. Importantly, higher response rates (both ORR and ≥VGPR), greater survival benefits, and longer TTNT were observed when DARA was initiated in earlier lines of therapy.

In comparison with the first interim analysis, in the overall population, an increase was observed for both ORR (71.8% to 74.1%) and the rate of ≥VGPR (51.4% to 55.6%) [14]. Moreover, the observed responses to DARA-based regimens seen here, with longer follow-up, are comparable with those observed in other real-world studies [15, 16]. At the prespecified interim analysis of the global phase 3 POLLUX RCT, which was done at a comparable follow-up time to the current study (median follow-up, 13.5 months for POLLUX vs. 16.2 months for the current study), patients with RRMM treated with DARA + IMiD + dexamethasone (specifically D-Rd) achieved an ORR of 92.9% and ≥VGPR rate of 75.8% [17], whereas patients treated with DARA + IMiD ± dexamethasone in the current study achieved an ORR of 76.1% and ≥VGPR rate of 58.2%. In the phase 3 LEPUS RCT (median follow-up, 8.2 months) among Chinese patients with RRMM treated with DARA + PI + dexamethasone (specifically D-Vd), rates of ORR and ≥VGPR were 82.5% and 65.0%, respectively [18], whereas patients treated with DARA + PI ± dexamethasone in the current study achieved rates of 73.1% and 55.8%. In light of inherent differences between RCTs and real-world studies (e.g., more frequent and compliant disease evaluations in RCTs), responses rates observed here in the MMY4032 study are generally comparable with RCTs of a similar patient population.

Survival outcomes observed in this real-world study are generally comparable with those of other real-world analyses [15] As seen with response rates, survival outcomes observed in MMY4032 were generally comparable with those reported for RCTs. In the LEPUS and CASTOR studies, 12-month PFS rates with D-Vd were 62.4% and 60.7%, respectively [18, 19], while in the current study among patients treated with DARA + PI ± dexamethasone, the 12-month PFS rate was 80.6%. Among patients receiving D-Rd in the POLLUX study, the 12-month PFS rate was 83.2% [17], whereas the 12-month PFS rate was 74.3% among patients treated with DARA + IMiD ± dexamethasone in the current real-world analysis. From subgroup analyses of current study, we found out that the early use of DARA would bring clear PFS benefits compared with late use. Meanwhile given the small sample size within each regimen subgroup at the certain lines, there was a limitation to explore the comparative effectiveness of individual DARA‑based regimens at the line groups. PFS and OS were similar regardless of age or revised cytogenetic risk status, and PFS and OS were prolonged in patients who received a longer versus shorter duration of therapy, highlighting the importance of continuous DARA treatment.

Many patients with MM, however, will still require subsequent therapy. In the current study, 24.1% of patients reported receiving subsequent non-DARA therapy. The prolonged TTNT observed with DARA-based regimens is also supported by observations from global clinical trials [11, 20, 21]. However, data on preferred subsequent therapies after DARA treatment and their associated outcomes remain limited in Chinese patients with MM, highlighting the need for further investigation. The current study provides valuable real-world insight into the regimens most commonly selected by health care providers, as well as the primary reasons for transitioning to subsequent treatments following DARA exposure in Chinese clinical practice. These findings further underscore the ongoing need for greater standardization of MM treatment strategies across hospitals in China.

Among the patients who underwent MRD testing, an overall MRD-negativity rate of 60.0% was observed across DARA-based regimens. DARA-based regimens that achieve superior clinical outcomes are often associated with deeper responses, such as MRD negativity, underscoring the importance of MRD negativity in MM [22, 23]. Real-world data on MRD negativity in patients with MM are relatively scarce, with most of these data coming from clinical trials [23, 24]. For instance, in patients with RRMM treated with DARA-based regimens, clinical trials such as POLLUX and LEPUS reported MRD negativity in 22.4% and 22.0% of patients in the intent-to-treat population at a median follow-up of 13.5 months and 8.2 months, respectively [17, 18]. Direct comparisons between real-world studies and RCTs are inappropriate, particularly as the MRD-negativity rate reported in this study was among patients who completed MRD testing while rates in RCTs are generally among the intent-to-treat population. Real-world studies face several challenges in assessing MRD negativity, including cost, testing capabilities, and variability in MRD detection methods and testing protocols, which can impact the comparability of MRD-negative rates with those reported in clinical trial settings [24–26]. Notably, the MRD testing method and interval of MRD testing were not standardized in this study, and flow cytometry—used with variable sensitivity—was the most common test method. Despite this heterogeneity, the MRD‑negativity rates observed still indicate that DARA‑based regimens achieved deep clinical responses in the real‑world setting.

One key consideration when making treatment decisions is the line of treatment in which therapies are initiated. In a US-based, real-world retrospective chart review study, treatment response rates were higher when DARA-based regimens were introduced in earlier treatment lines [15]. Findings from the first interim analysis, as well as the consistently observed results in the current MMY4032 analysis, further support this observation. Higher response rates and improved 12‑month PFS and OS rates were seen when daratumumab was initiated in the first‑ or second‑line settings compared with later‑line settings. In addition, a longer duration of daratumumab exposure was associated with more favorable PFS and OS outcomes compared with shorter treatment. These trends in effectiveness and duration of exposure support the early and longer use of DARA-based treatment for MM.

The safety profile remained consistent with that previously reported, with no new safety concerns despite the increase in median DARA exposure observed with longer follow-up. Rates of ADRs and serious TEAEs increased slightly from the first interim analysis (ADRs, 18.9% vs. 20.3%; serious TEAEs, 13.7% vs. 15.6%). The rate of serious TEAEs observed here remains much lower than that reported in some pivotal MM RCTs, such as the LEPUS (D-Vd), OCTANS (D-VMP), and POLLUX (D-Rd) studies (15.6% vs. 48.6%, 43.8%, and 48.8%, respectively) [12, 17, 18]. This disparity may be due to some inevitable differences between study designs. For example, in real-world studies, doctor visits may be less frequent, TEAE reporting may be inconsistent between centers, and patients may have reduced awareness or willingness to report TEAEs. Finally, with longer follow-up, the number of deaths due to TEAEs remained low (*n* = 8), with only 1 TEAE leading to death considered DARA related (acute-onset chronic liver failure).

While the current study, like other real-world studies, is limited by the variability in the completeness of patient medical charts and electronic case report forms at each site, the real-world data presented here are supported by findings from RCTs. Moreover, data from the current study were collected from multiple centers across varying regions of China, including hospitals in which a significant number of patients with MM are treated; thus, this broad representation further reinforces the clinical benefit of DARA-based MM regimens as reported in this study.

In conclusion, this updated analysis of the large, real-world, observational MMY4032 study continues to provide vital insight into treatment decisions in routine clinical practice for Chinese patients with MM. Response rates continued to deepen with additional follow-up, survival rates remained high and generally consistent across DARA-regimens, MRD negativity was achieved in more than half of patients who underwent testing, and the estimated proportion of patients free from next line of treatment at 12 months was > 80%. Outcomes were most favorable when DARA was initiated in earlier lines of therapy. No new safety concerns were identified, and the overall safety profile of DARA-based regimens remained comparable with that previously described in RCTs. Overall, data from this updated analysis continue to support the early and continuous use of DARA-based regimens as a standard of care in Chinese patients with MM.

## Supplementary Information

Supplementary Material 1.

## Data Availability

All relevant data for this analysis are included in the manuscript and supplemental files.
