# Supplementary material for: Treatment patterns, effectiveness, and safety of daratumumab-based regimens in Chinese patients with multiple myeloma: longer follow-up of the real-world MMY4032 study
Source: Ann Hematol. 2026 Apr 13;105(5):240. doi: 10.1007/s00277-026-06972-8 (PMC13070976; doi:10.1007/s00277-026-06972-8)
Supplement: Supplementary file 1 — Supplementary Material 1. [file 277_2026_6972_MOESM1_ESM.docx]

# Supplementary Appendix

Supplement to *Annals of Hematology article*: Treatment Patterns, Effectiveness, and Safety of Daratumumab-Based Regimens in Chinese Patients With Multiple Myeloma: Longer Follow-up of the Real-World MMY4043 Study

Wei Yang, Luqun Wang, Yafei Wang, Ting Niu, Rong Fu, Yuping Zhong, Wenbin Qian, Kaiyang Ding, Kai Sun, Hong Liu, Baijun Fang, Hui Liu, Yanhui Li, Yishen Yang, Jianmin Zhuo, Xi Chen, Bijie Xun, Jin Lu

**Corresponding author:**

Jin Lu

Peking University People’s Hospital, National Clinical Research Center for Hematologic Disease, Beijing, China; Collaborative Innovation Center of Hematology, Soochow, China Email: jin1lu@sina.com

**Supplementary Methods**

## Primary and secondary objectives

The primary objective of the real-world MMY4032 study was to describe treatment patterns and clinical outcomes in routine clinical practices among patients with multiple myeloma (MM) treated with daratumumab (DARA) in China. The secondary objective was to assess the safety and tolerability of DARA in Chinese patients with MM.

To assess clinical outcomes and clinical response measures, treatment response was evaluated in accordance with the International Myeloma Working Group (IMWG) 2016 response criteria. Key clinical outcomes/clinical response parameters explored in MMY4032 were as follows:

Overall response rate (ORR), defined as the proportion of patients who had a best response of complete response (CR), very good partial response (VGPR), or partial response (PR) during daratumumab-based treatment

≥VGPR, defined as proportion of patients who have a very good partial response or better (stringent complete response [sCR], CR, VGPR) during the daratumumab-based therapies

Response rates (as per IMWG criteria)

Progression-free survival (PFS), defined as the length of time from the index date (the date of initiation of first dose of daratumumab) until the first documented disease progression or death due to any cause, whichever comes first, during the daratumumab-based therapies

Time to next treatment (TTNT), defined as the interval from the date of daratumumab initiation to the date of commencement of the next line of treatment due to lack of efficacy, disease progression, or death

Overall survival (OS), defined as the time from the index date to the date of death, for any cause of the patient’s death

**Supplementary Table 1. Patient demographic and baseline disease characteristics^a^**

| **Characteristic** | **Overall  (N = 212)** | **DARA monotherapy  (n = 24)** | **DARA + dexa (n = 20)** | **DARA + PI ± dexa (n = 61)** | **DARA + IMiD ± dexa (n = 73)** | **DARA + PI + IMiD ± dexa (n = 28)** | **DARA + other agents  (n = 6)** |
| --- | --- | --- | --- | --- | --- | --- | --- |
| Age, median (range), y |  |  |  |  |  |  |  |
| At MM diagnosis | 61 (29-89) | 61.5 (43-81) | 62 (42-83) | 62 (37-89) | 61 (29-81) | 59.5 (41-74) | 55 (43-66) |
| At baseline | 64 (29-89) | 66 (46-81) | 64.5 (42-83) | 63 (37-89) | 65 (29-82) | 59.5 (41-76) | 56.5 (46-69) |
| Time from diagnosis to DARA initiation |  |  |  |  |  |  |  |
| n | 211 | 24 | 20 | 61 | 72 | 28 | 6 |
| Median (range), y | 1 (0-12) | 2 (0-8) | 1 (0-9) | 0 (0-6) | 1.5 (0-12) | 0 (0-12) | 2 (0-5) |
| Sex, n (%) |  |  |  |  |  |  |  |
| Male | 122 (57.5) | 13 (54.2) | 10 (50.0) | 42 (68.9) | 39 (53.4) | 14 (50.0) | 4 (66.7) |
| Female | 90 (42.5) | 11 (45.8) | 10 (50.0) | 19 (31.1) | 34 (46.6) | 14 (50.0) | 2 (33.3) |
| ISS disease stage, n (%) |  |  |  |  |  |  |  |
| n | 141 | 15 | 12 | 36 | 55 | 20 | 3 |
| I | 30 (21.3) | 3 (20.0) | 2 (16.7) | 3 (8.3) | 16 (29.1) | 6 (30.0) | 0 |
| II | 53 (37.6) | 7 (46.7) | 6 (50.0) | 11 (30.6) | 19 (34.5) | 9 (45.0) | 1 (33.3) |
| III | 58 (41.1) | 5 (33.3) | 4 (33.3) | 22 (61.1) | 20 (36.4) | 5 (25.0) | 2 (66.7) |
| ECOG PS, n (%) |  |  |  |  |  |  |  |
| n | 131 | 17 | 12 | 36 | 44 | 18 | 4 |
| 0 | 30 (22.9) | 3 (17.6) | 2 (16.7) | 10 (27.8) | 12 (27.3) | 2 (11.1) | 1 (25.0) |
| 1 | 76 (58.0) | 9 (52.9) | 6 (50.0) | 20 (55.6) | 24 (54.5) | 14 (77.8) | 3 (75.0) |
| 2 | 16 (12.2) | 4 (23.5) | 3 (25.0) | 3 (8.3) | 4 (9.1) | 2 (11.1) | 0 |
| ≥3 | 9 (6.9) | 1 (5.9) | 1 (8.3) | 3 (8.3) | 4 (9.1) | 0 | 0 |
| Revised cytogenetic risk,^b^ n (%) |  |  |  |  |  |  |  |
| n | 76 | 3 | 7 | 22 | 29 | 12 | 3 |
| High-risk^c^ | 40 (52.6) | 2 (66.7) | 5 (71.4) | 12 (54.5) | 14 (48.3) | 7 (58.3) | 0 |
| t(4;14) | 10 (13.2) | 0 | 1 (14.3) | 4 (18.2) | 3 (10.3) | 2 (16.7) | 0 |
| t(14;16) | 3 (3.9) | 0 | 0 | 1 (4.5) | 2 (6.9) | 0 | 0 |
| del(17p) | 9 (11.8) | 0 | 1 (14.3) | 0 | 6 (20.7) | 2 (16.7) | 0 |
| gain(1q21) | 20 (26.3) | 1 (33.3) | 3 (42.9) | 6 (27.3) | 7 (24.1) | 3 (25.0) | 0 |
| amp(1q21) | 12 (15.8) | 1 (33.3) | 1 (14.3) | 4 (18.2) | 5 (17.2) | 1 (8.3) | 0 |
| t(14;20) | 4 (5.3) | 0 | 1 (14.3) | 1 (4.5) | 1 (3.4) | 1 (8.3) | 0 |

DARA, daratumumab; dexa, dexamethasone; ECOG PS, Eastern Cooperative Oncology Group performance status; IMiD, immunomodulatory drug; ISS, International Staging System; MM, multiple myeloma; PI, proteasome inhibitor.

^a^Data from the safety population (defined as all enrolled patients who received ≥1 dose of daratumumab; patients were included in the analysis according to the dose received).

^b^Cytogenetic abnormalities were based on reported fluorescence in situ hybridization results.

^c^Revised high cytogenetic risk was defined as ≥1 of the following high-risk cytogenetic abnormalities: t(4;14), t(14;16), del(17p), t(14;20), gain(1q21), and amp(1q21).

**Supplementary Table 2. DARA treatment duration and exposure by overall population**

|  | **Overall  (N = 212)** |
| --- | --- |
| Number of DARA cycles |  |
| Mean (SD) | 6.6 (5.4) |
| Median (range) | 5.0 (1.0-26.0) |
| Total duration of DARA exposure, mo |  |
| Mean (SD) | 10.4 (8.7) |
| Median (range) | 8.2 (0-49.1) |
| Duration of DARA exposure in first line, mo |  |
| n | 35 |
| Mean (SD) | 12.2 (9.5) |
| Median (range) | 9.7 (0.3-37.0) |
| Duration of DARA exposure in second line, mo |  |
| n | 115 |
| Mean (SD) | 10.1 (8.5) |
| Median (range) | 8.1 (0-49.1) |
| Duration of DARA exposure in third line, mo |  |
| n | 33 |
| Mean (SD) | 10.3 (9.2) |
| Median (range) | 7.5 (0.1-31.5) |
| Duration of DARA exposure in fourth line, mo |  |
| n | 29 |
| Mean (SD) | 9.5 (8.8) |
| Median (range) | 4.7 (0.5-28.7) |

DARA, daratumumab; SD, standard deviation.

**Supplementary Fig. 1 Overall response rate in patient subgroups^a^**

**
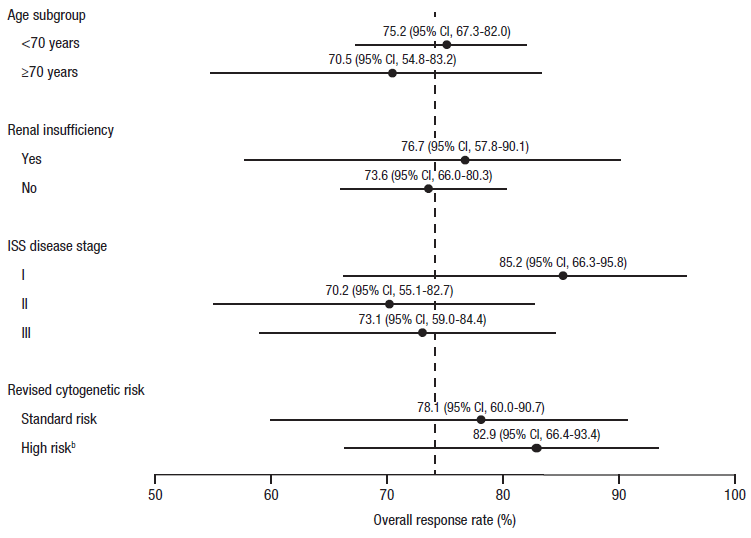
**

The vertical dashed line indicates the overall response rate in the response-evaluable population, 74.1%.

CI, confidence interval; DARA, daratumumab; ISS, International Staging System.

^a^In patients with available post-DARA disease assessment.

^b^Revised high cytogenetic risk was defined as ≥1 of the following high-risk cytogenetic abnormalities: t(4;14), t(14;16), del(17p), t(14;20), gain(1q21), and amp(1q21).

**
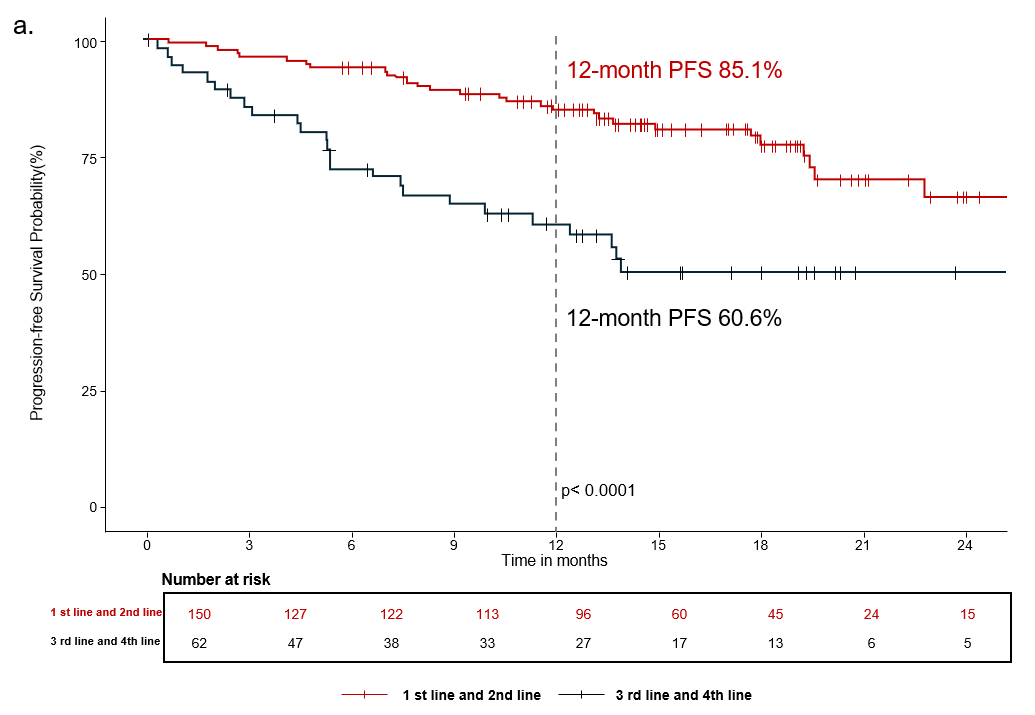
Supplementary Fig. 2 PFS in patient subgroups by a) early (1^st^ line and 2^nd^ line) /late line (3^rd^ line and 4^th^ line) in which DARA was initiated and b) by DARA-based regimen in early line**

**
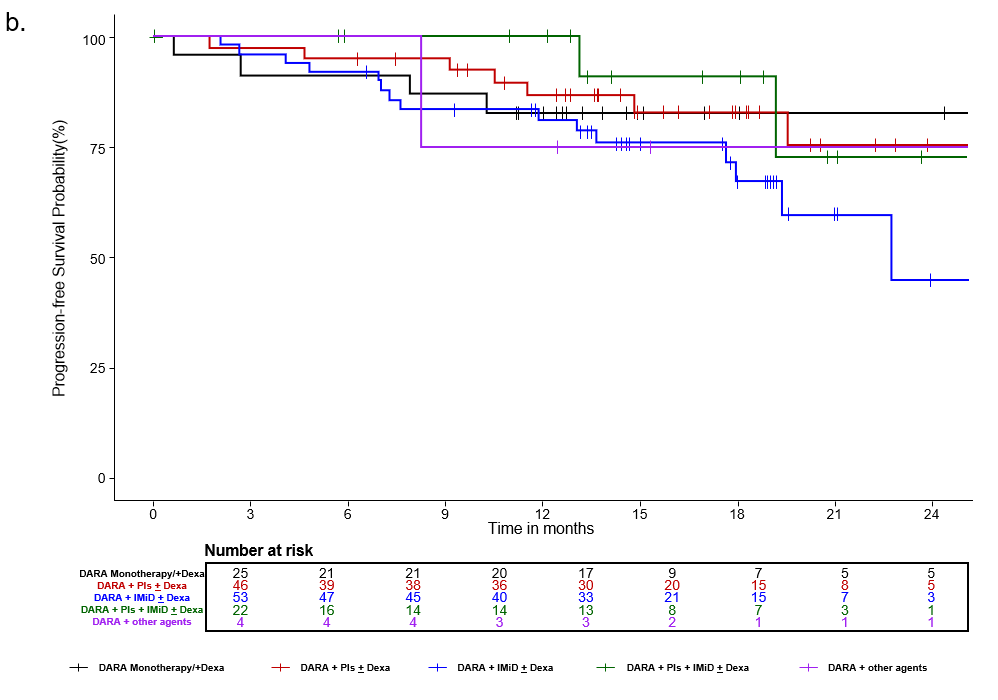
**

**Supplementary Fig. 3 PFS in patient subgroups by a) age (70 vs ≥70 years), b) revised cytogenetic risk (standard vs high^a^), c) treatment duration (<12 vs ≥12 months), and d) treatment duration (<18 vs ≥18 months)**

**
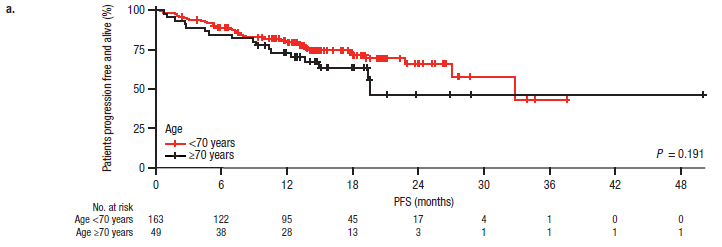
**

**
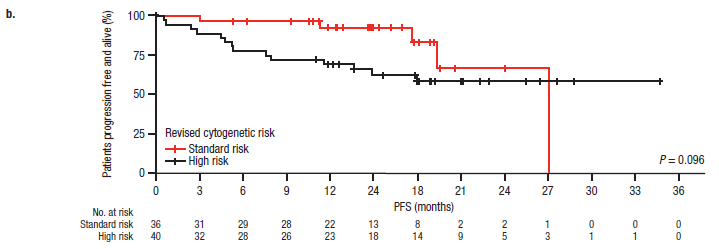
**

**
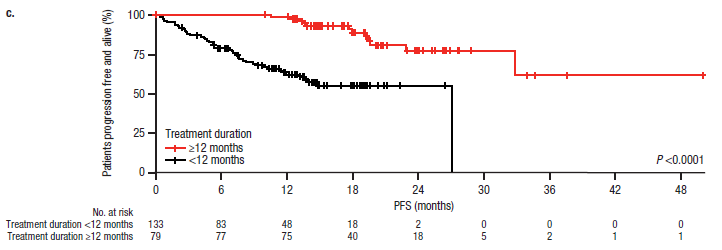
**

**
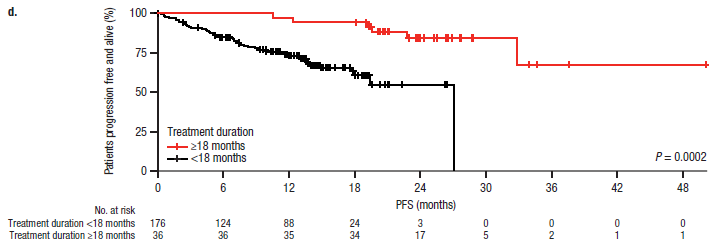
**

PFS, progression-free survival.

^a^Revised high cytogenetic risk was defined as ≥1 of the following high-risk cytogenetic abnormalities: t(4;14), t(14;16), del(17p), t(14;20), gain(1q21), and amp(1q21).

**Supplementary Fig. 4 OS in patient subgroups a) age (70 vs ≥70 years), b) revised cytogenetic risk (standard vs high^a^), c) treatment duration (<12 vs ≥12 months), and d) treatment duration (<18 vs ≥18 months)**

**
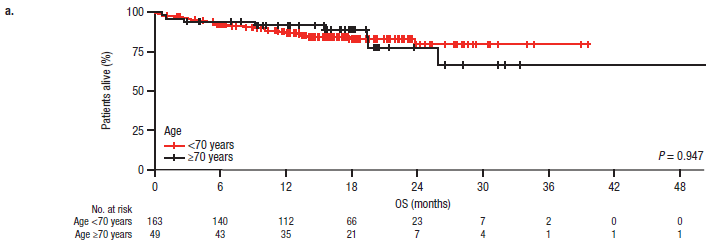
**

**
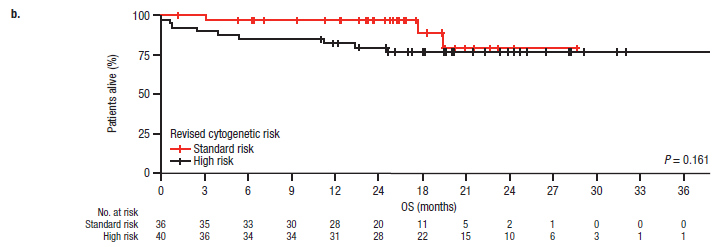
**

**
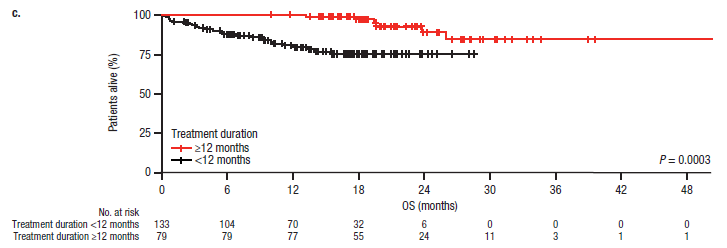
**

**
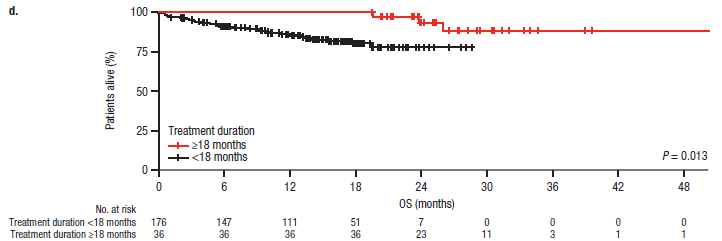
**

OS, overall survival.

^a^Revised high cytogenetic risk was defined as ≥1 of the following high-risk cytogenetic abnormalities: t(4;14), t(14;16), del(17p), t(14;20), gain(1q21), and amp(1q21).
